# Case Report: Obstetric and COVID-19-Related Morbidity and Mortality in Three Patients with Sickle Hemoglobinopathy

**DOI:** 10.4269/ajtmh.23-0761

**Published:** 2024-04-23

**Authors:** Shanea Gibson, Tiffany Hunter, Nadine Johnson

**Affiliations:** ^1^ Department of Obstetrics and Gynaecology, University Hospital of the West Indies, Kingston, Jamaica;; ^2^ Department of Obstetrics and Gynaecology, University of the West Indies, Kingston, Jamaica

## Abstract

Approximately 3% of pregnant women have sickle cell disease (SCD). COVID-19, caused by severe acute respiratory syndrome coronavirus 2 (SARS-CoV-2), became a global pandemic in March 2020, resulting in more than 3,500 deaths in Jamaica by April 2023. Sickle cell disease is an immunocompromised state; therefore, contracting COVID-19 may result in adverse maternal/neonatal outcomes. Current literature focusing on individuals of Afro-Caribbean descent is limited. Our objective was to describe the obstetric and neonatal outcomes of pregnant patients with SCD who contracted COVID-19. A retrospective case series was conducted at the University Hospital of the West Indies (Jamaica) from 2020 to 2022. We describe the maternal and neonatal outcomes of three patients with COVID-19 and SCD (including two with hemoglobin SC disease and one with hemoglobin SS disease), with complications including the demise of a mother and a newborn. Vaso-occlusive crisis was the more common presentation. Two patients required ventilatory support. Although previous reports have shown similar clinical sequelae in pregnant and nonpregnant patients with SCD and COVID-19, maternal and neonatal deaths remain possible.

## INTRODUCTION

Sickle cell disease (SCD) is the most common inherited hemoglobinopathy predominantly affecting persons of African ancestry, resulting in a reduced erythrocyte life span and chronic hemolytic anemia.^1^ In the Jamaican pregnant population, 2.8% of women have SCD.^2^ This group of disorders is highly associated with increased maternal morbidity/mortality,^3^ with the most common cause of maternal death being acute chest syndrome (ACS).^2^

COVID-19, caused by severe acute respiratory syndrome coronavirus 2 (SARS-CoV-2), was officially declared a pandemic in March 2020. Patients with chronic medical illnesses are prone to severe disease/mortality.^4^ Antepartum women are more susceptible to severe pneumonia owing to the physiological changes in pregnancy, namely impaired immunity, reduced respiratory reserves, and hypercoagulability coupled with increased cardiac demand.^5^ These changes are further aggravated in SCD patients, resulting in vaso-occlusive crisis (VOC), ACS, and venous thromboembolic events.^6^

In the era of the COVID-19 pandemic, it is immensely important to highlight the various outcomes observed with an uncommon condition in the obstetric patient.^6^ Prior case studies/series have been reported in Brazil,^7^ India,^6^ and Oman.^8^ Outcomes were generally favorable, concluding that pregnant patients with SCD and COVID-19 infection had courses similar to those of the nonpregnant SCD population.^8^ Jamaica is a low- to middle-income emerging economy with limited resources for support of the critically ill. Here, we report a case series of COVID-19 infection in pregnant patients with SCD and highlight their obstetric outcomes. Ethical approval was obtained from the University of the West Indies–Mona Campus, Jamaica (CREC-MN.0269, 2022/2023).

## CASE 1

A 29-year-old gravida 2, para 1 (G2P1) (hemoglobin SC disease [HbSC]; steady-state hemoglobin, 10.9 g/dL) had an uneventful antenatal period. At gestational age 36 weeks and 6 days, she presented complaining of lower back pain for 1 day. Vital signs revealed fever (38°C) and new-onset hypertension of 151/87 mm of Hg without proteinuria or symptoms of preeclampsia. The COVID-19 polymerase chain reaction (PCR) test was positive. She received metamizole (analgesia/antipyretic) with intravenous hydration and then α-methyldopa (antihypertensive) and was subsequently discharged after 3 days. She returned 3 days later with worsening joint pains; however, all vital signs were within normal limits. Paracetamol/codeine was administered for analgesia, followed by misoprostol induction at gestation age 37 weeks and 6 days, with subsequent vaginal delivery 17 hours later. She was discharged 2 days postdelivery with her neonate.

## CASE 2

A 31-year-old gravida 3, para 1 and 1 miscarriage (G3P1+1) (HbSC; steady-state hemoglobin, unsure) with an uneventful antenatal period presented at 37 weeks and 3 days complaining of joint pain and was assessed as having VOC. She was afebrile but tachycardic at 117 beats per minutes and tachypneic at 30 breaths per minute, maintaining oxygen saturation (SpO_2_) of 98% on room air. Fetal tachycardia was noted and was resolved with intrauterine resuscitation. The COVID-19 PCR test was positive. Opioid analgesia was administered, and there were no abnormal respiratory findings on auscultation. The maternal vitals normalized once the patient’s pain was controlled. She was induced and delivered 16 hours later after vacuum-assisted delivery for fetal malposition. Immediately postpartum, she became febrile at 38.5°C with a respiratory rate of 18 breaths per minute and an SpO_2_ of 95% on 5 L/minute oxygen therapy via face mask. The neonate was admitted because of low-grade pyrexia. Antipyretics were administered, and the respiratory examination remained unremarkable. Vaso-occlusive crisis returned, and the patient became tachypneic and tachycardic 3 hours postdelivery with harsh breath sounds throughout. She was assessed as having COVID-19 pneumonia and ACS and administered remdesivir, dexamethasone, therapeutic unfractionated heparin, and paracetamol. Her oxygen requirements increased rapidly, which led to transfer to the intensive care unit (ICU), intubation, and ventilation. Hypotension and bradycardia were noted, and she was started on inotropes. However, cardiopulmonary arrest ensued with no return of spontaneous circulation after 40 minutes of advanced cardiopulmonary resuscitation.

## CASE 3

A 23-year-old primigravida (hemoglobin SS disease [HbSS]; steady-state hemoglobin, 6 g/dL) at gestational age 28 weeks and 6 days was transferred from a rural hospital for chest pain, fever, and worsening dyspnea. Her last sickle crisis was in childhood, requiring blood transfusion. At the referral center, she was febrile at 40.5°C and tachypneic at 40 breaths per minute with an SpO_2_ of 88% on room air, which improved with 5 L/minute of oxygen. She became hypotensive, requiring noradrenaline infusion, and commenced ceftriaxone, which was switched to piperacillin/tazobactam. Upon transfer, frank hematuria was noted with occasional hypoglycemic episodes. The urea was elevated at 9.2 mmol/L with a deranged coagulation profile and transaminases. Her oxygen requirements increased to 10 L/minute via face mask. She was transfused fresh-frozen plasma to correct her coagulopathy and dextrose infusion to maintain normoglycemia. She was assessed as having acute fatty liver in pregnancy (AFLP) and was delivered via emergency Cesarean section under general anesthesia at 29 weeks. Intraoperatively, the blood loss was 350 mL, and she continued to bleed postoperatively despite uterotonic agents, bimanual uterine compression, and intrauterine balloon tamponade. After an estimated blood loss of 1.15 L with ongoing hemorrhage unresponsive to conservative management, an emergency subtotal hysterectomy was performed. She was transferred to the ICU and extubated on day 2 postoperatively. Antibiotics were changed to meropenem, and packed red blood cells were transfused. She was discharged from the ICU on day 4 postoperatively. Urine, vaginal, and blood cultures were sterile. On day 11 postdelivery, she was transferred to the referral center to complete parenteral antibiotics (see Supplemental Table 1).

## DISCUSSION

We report three cases of COVID-19 infection in pregnant patients with SCD at various gestational ages. It has been found that in nonpregnant patients, SCD may have a protective effect against severe COVID-19 clinical sequelae because of the background chronic inflammation, hemolysis, and anemia.^9^ At baseline, SCD patients have elevated cytokines, possibly protecting them against the cytokine storm associated with severe COVID-19 pneumonia.^10^

Prior case studies/series have been reported in other countries^6^^–^^8^ with generally favorable outcomes.^8^ Kolanska et al.^11^ conducted a retrospective cohort study in France of eight pregnant women with SCD and COVID-19 and concluded that COVID-19 rarely triggered a crisis and that fetal outcomes were positive. COVID-19 is associated with ACS,^10^ which is typically triggered by bacterial infection,^7^ in addition to bone marrow fat embolization and intravascular pulmonary sequestration.^12^

In our study, two of the three women (both with HbSC) presented without respiratory symptoms. Other case reports have shown that the most common symptoms were VOC and fever,^9^^,^^13^ with none describing a maternal mortality. Interestingly, our maternal death was the only case that presented at term, with worsening symptoms in the immediate postpartum period. A computer tomography pulmonary angiogram was desirable, but she was too unstable to be transported to radiology. She was aggressively ventilated in the ICU. Extracorporeal membrane oxygenation may have been useful^14^ but is unfortunately not a part of our armamentarium.

Hardy et al.^10^ described an HbSC case with respiratory symptoms 2 weeks postdelivery and possible hyperhemolysis syndrome, although oxygen or ICU care was not indicated. It was further suggested that individuals with the SS genotype experience a more severe presentation, but an antenatal population was not studied.^10^ Gowri et al.^8^ described one HbSC obstetric patient, but she miscarried in the first trimester with a hydatidiform mole.

Arlet et al.^15^ examined all SCD patients who were confirmed COVID-19 RNA positive in France and reported two deaths from COVID-19 pneumopathy (both men, with HbSC, and between 45 and 64 years old). In this study, 63% of patients with the HbSC genotype were admitted to the ICU, compared with 17% of those with the SS/Sβ° genotype (*P* <0.05), and thus patients with the HbSC genotype may display a more severe course.^15^ Kolanska et al.^11^ further examined this possibility and concluded that the clinical course in pregnant women with SCD is not affected by the genotype.

In case 3, the diagnosis of AFLP was made based on Swansea criteria^16^ in accordance with evidence of coagulopathy, leukocytosis, acute kidney injury, elevated alanine transaminase, elevated bilirubin, and hypoglycemia. COVID-19 has been described as the great imitator,^17^ and thus hepatic impairment, seen in 19.5% of patients,^18^ may have resulted from this. Ideally, maternal liver biopsy or newborn testing for long-chain 3-hydroxyacyl coenzyme A dehydrogenase deficiency may confirm this diagnosis.^19^^,^^20^ Neonatal metabolic screening is not locally accessible, and maternal delivery was expedited in lieu of further investigations because of uncorrected coagulopathy and limited availability of blood products. Histopathology, such as placental in all three cases, lung biopsy for case 2, and liver biopsy for case 3, may have been helpful. Unfortunately, our laboratory was not equipped to safely process potentially infectious specimens from COVID-19 patients.

With both pregnancy and SCD being considered immunosuppressed states, secondary to immunomodulation to prevent fetal rejection and asplenia, respectively, the additional effect of a superimposed viral infection that resulted in over 600 million deaths worldwide should be examined. There is a paucity of reports detailing the effects of COVID-19 infection in this patient population. The relatively short history of the COVID-19 pandemic further influences available data, recommendations, and the institution of any specific guidance to care for patients when these two conditions meet.^6^

To the best of our knowledge, this serves as the first case series to be reported in an Afro-Caribbean populace of a low- to middle-income country with limited medical resources. In the era of pandemics with emerging novel microorganisms that have adverse clinical effects on humans, it is useful to report our experiences and outcomes. The effects of COVID-19 infection in pregnant women with underlying comorbidities is of particular interest. The creation of a registry may further enhance knowledge.^7^ It may also be useful to examine the effect of COVID-19 vaccination on infection incidence and severity in this population.

## Supplemental Materials

10.4269/ajtmh.23-0761 Supplemental Materials
