# Supplementary material for: Case Report: Obstetric and COVID-19-Related Morbidity and Mortality in Three Patients with Sickle Hemoglobinopathy
Source: Am J Trop Med Hyg. 2024 Apr 23;110(6):1198–200. doi: 10.4269/ajtmh.23-0761 (PMC11154032; doi:10.4269/ajtmh.23-0761)
Supplement: Supplemental Materials [file tpmd230761.SD1.pdf]

**Table S1:** Maternal and neonatal laboratory investigations and clinical features			
**Maternal demographics**			
Parameters	Case 1	Case 2	Case 3
Age/years	29	31	23
SCD Genotype	SC	SC	SS
Antenatal drug history (prior to COVID-19 infection)	Low-dose aspirin, folic acid	Low-dose aspirin, folic acid	Low-dose aspirin, folic acid
Echocardiogram done in mid-trimester	Normal findings	Normal findings	Normal findings
**COVID-19 clinical course**			
Presenting symptoms	VOC	VOC	Chest pain, fever, dyspnoea,
Initial Temperature (℃)	38.0	37.3	40.5
Initial RA SPO2	95%	98%	87 – 90%
GA at diagnosis	36^{+6}	37^{+3}	28^{+5}
GA at delivery	38^{+0}	37^{+4}	29^{+0}
ICU Admission	No	Yes	Yes
Length of stay	First admission – 3 days; Readmission – 5 days	3 days	11 days
COVID severity	Mild	Severe	Severe
ACS	No	Yes	Yes
Other complications	PIH	NIL	AFLP, AKI, PPH
Therapy received	LMWH, metamizole, paracetamol/ codeine	Remdesivir, dexamethasone, paracetamol	Piperacillin/tazobactam → meropenem, dexamethasone, LMWH, azithromycin,
Transfusions in pregnancy	No	No	Yes, Simple
Steady state Hb (g/dl)	10.9	Unknown	6.0
Nadir Hb (g/dl)		8.9	5.2
WBC at presentation ($*10^9$)	13.7	18.8	27.3
Highest WBC ($*10^9$)		31.1	91.4
COVID-19 Vaccine status	Not received	Not received	Not received
Non-stress test	First admission –	On admission –	Category 2 – minimal

	Category 1<br><br>Re-admission – Category 1	Category 2<br><br>Post intra-uterine resuscitation and intrapartum – category 1	variability (less than 5 beats per minute over 40 minutes)
Intrapartum fetal monitoring	Continuous EFM	Continuous EFM	Delivered via Caesarean section
**Neonatal outcome**			
Parameters	Case 1	Case 2	Case 3
Birth weight	3.82 kg	3.41 kg	1.0 kg
5-min APGAR	9	9	1
SCN admission	No	Yes	Yes
NND	No	No	Yes

Abbreviations: ACS – acute chest syndrome; AFLP – acute fatty liver of pregnancy; AKI – acute kidney injury; EFM – electronic fetal monitoring; GA – gestational age; Hb – hemoglobin; ICU – intensive care unit; LMWH – low-molecular weight heparin; NND – neonatal death; PIH – pregnancy induced hypertension; PPH – postpartum hemorrhage; RA – room air; SCN – special care nursery; VOC – vaso-occlusive crisis; WBC – white blood cell
